# The complex lipidome as a driver of tissue-specific pathology in adrenoleukodystrophy

**DOI:** 10.1186/s13024-026-00975-9

**Published:** 2026-07-17

**Authors:** Stephan Kemp, Marc Engelen, Yorrick R. J. Jaspers

**Affiliations:** 1 Laboratory Genetic Metabolic Diseases, Department of Laboratory Medicine, Amsterdam Neuroscience, Amsterdam UMC Location University of Amsterdam, Amsterdam Gastroenterology Endocrinology Metabolism, Amsterdam, The Netherlands; 2 Department of Pediatric Neurology, Amsterdam Leukodystrophy Center, Amsterdam UMC Location University of Amsterdam, Emma Children’s Hospital, Amsterdam Neuroscience, Amsterdam, The Netherlands; 3 Laboratory Genetic Metabolic Diseases (F0-132), Department of Laboratory Medicine, Amsterdam UMC location University of Amsterdam, Meibergdreef 9, Amsterdam, 1105AZ The Netherlands

**Keywords:** X-linked adrenoleukodystrophy, Very-long-chain fatty acids, Lipidomics, Lysophosphatidylcholine, Leukodystrophy, Myelopathy, Adrenal insufficiency, ELOVL1, Neuroinflammation, Substrate reduction therapy

## Abstract

X-linked adrenoleukodystrophy (ALD) is an inherited peroxisomal disorder caused by pathogenic variants in the *ABCD1* gene, encoding a peroxisomal membrane transporter required for the import of very-long-chain fatty acids (VLCFA) into peroxisomes for degradation. ABCD1 deficiency leads to VLCFA accumulation in plasma and tissues. The resulting disease has a highly variable clinical presentation. In males, this manifests as cerebral demyelination, progressive myelopathy, and adrenal insufficiency, alone or in combination. Women predominantly develop myelopathy, while cerebral disease and adrenal insufficiency are rare, occurring almost exclusively in cases of extreme X-inactivation skewing toward the mutant allele. The lipid-mediated mechanisms linking VLCFA accumulation to tissue-specific pathology remain incompletely understood. Here, we review evidence that VLCFA-containing complex lipids, rather than free VLCFAs alone, are central mediators of tissue-specific pathology in ALD and discuss the therapeutic implications of this lipid-centric perspective. VLCFAs are incorporated into a broad range of complex lipids, including phosphatidylcholines, lysophosphatidylcholines, cholesterol esters, triacylglycerols, sphingomyelins, ceramides, and plasmalogens. The degree of lipid dysregulation increases with acyl chain length and saturation. VLCFA-containing lipid species correlate with disease severity across all clinical phenotypes. In the brain, VLCFA-containing phosphatidylcholines accumulate before demyelination onset, cholesterol ester accumulation is associated with neuroinflammatory cascades, and plasmalogen depletion reflects early oxidative damage. In the spinal cord, VLCFA-containing myelin lipids are associated with non-inflammatory axonopathy, mitochondrial dysfunction, and microglial phagocytic activation. In the adrenal gland, VLCFA accumulation in cholesterol ester-rich lipid droplets impairs ACTH receptor signaling and sequesters cholesterol from steroidogenic pathways. Enzymatic regulators of VLCFA homeostasis, including ELOVL1, SCD1, and the omega-oxidation enzymes CYP4F2 and CYP4F3B, are potential therapeutic targets for substrate reduction. Plasma VLCFA-lipid profiles correlate with disease severity across all affected tissues, positioning lipidomic profiling as a potential clinical instrument for risk stratification and treatment monitoring. Secondary lipid mediators amplify primary VLCFA toxicity through distinct, cell-type-specific pathways. The near-exclusive occurrence of cerebral ALD and adrenal insufficiency in women with extreme X-inactivation skewing suggests that partial reduction of the VLCFA lipid burden, rather than complete normalization, may be sufficient to prevent severe disease manifestations. This has direct implications for substrate-reduction therapy development.

**Graphical abstract:**

**Figure:**
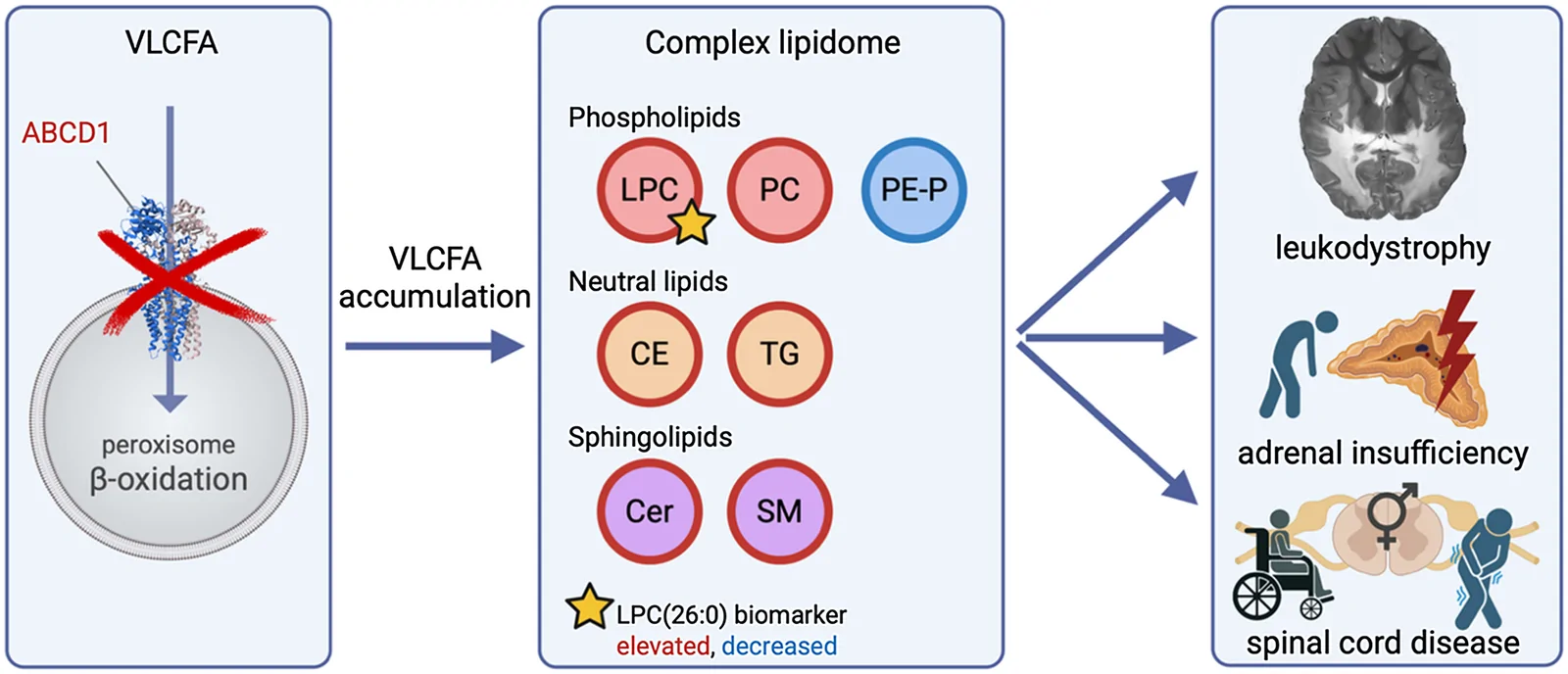

## Introduction

X-linked adrenoleukodystrophy (ALD; OMIM: 300100) was first described in 1923 by Siemerling and Creutzfeldt, who reported a boy with progressive skin discoloration and cerebral demyelination [1]. Its genetic basis was not established until 1993, when the *ABCD1* gene was identified [2]. ALD is caused by pathogenic variants in this gene, with an estimated incidence of approximately 1 in 17,000 births [3]. Located on the Xq28 region of the X chromosome, *ABCD1* spans 19.9 kilobases and comprises 10 exons [2]. To date, more than 1000 distinct pathogenic variants have been reported in the ABCD1 Variant Registry (www.adrenoleukodystrophy.info) [4, 5]. The *ABCD1* gene encodes a 745 amino acid transmembrane half-transporter of the ATP-binding cassette family, which plays a crucial role in the transport of coenzyme A-activated very-long-chain fatty acid (VLCFA) esters (≥ C22:0) into peroxisomes for degradation [6–8]. Impairment of ABCD1 protein function results in defective peroxisomal β-oxidation [9], leading to the accumulation of VLCFAs in plasma and tissues [10].

ALD has a highly variable and unpredictable clinical presentation in males. It primarily affects the white matter of the brain, the spinal cord, and the adrenal cortex [11]. The precise mechanism underlying this selective tissue involvement is unclear [12, 13]. Patients are asymptomatic at birth but develop symptoms as the disease progresses. Around 80% of male ALD patients will develop adrenal insufficiency, with about half of these cases presenting in the first decade of life [14]. This condition is characterized by elevated adrenocorticotropic hormone (ACTH) and low cortisol levels [15]. Although adrenal insufficiency can be managed with corticosteroid replacement therapy, it is often missed or overlooked due to the non-specific nature of its symptoms [16, 17]. If left undiagnosed and untreated, adrenal insufficiency can result in a life-threatening adrenal crisis.

All male ALD patients develop a progressive spinal cord disease. The age of onset and rate of progression vary significantly [18]. Symptoms typically manifest in the third decade of life and often appear as a slowly progressive gait disorder caused by spastic paraparesis and sensory ataxia. By the sixth decade of life, most patients require assistance walking or are wheelchair-bound. Accompanying symptoms commonly include neurogenic bladder dysfunction, incontinence, and sexual dysfunction. Currently, there is no disease-modifying treatment available.

Male patients with ALD have a 60% lifetime risk of developing cerebral ALD, a progressive leukodystrophy [14, 19, 20]. Cerebral ALD manifests in 30 to 40% of ALD patients between the ages of 3 and 18. Initial symptoms often present as cognitive impairment and behavioral changes, which are nonspecific and may lead to delayed diagnosis. As cerebral ALD progresses, overt neurological symptoms appear, such as visual agnosia, spastic tetraparesis, and epileptic seizures. In its advanced stages, cerebral ALD leaves patients bedridden, blind, and unresponsive. Without treatment, death usually occurs within two to four years of symptom onset. However, intensive care can prolong survival in a vegetative state for years. Hematopoietic stem cell transplantation (HCT) and gene therapy can halt the progression of cerebral ALD [21–24]; however, both must be performed in the early stages of the disease to be successful [16, 25]. Therefore, early diagnosis is of paramount importance to achieving favorable outcomes [26].

An increasing number of countries, including the United States, the Netherlands, and Taiwan, have implemented newborn screening programs [27–29]. These programs identify patients before symptoms appear and have become essential for timely intervention, including MRI surveillance and prompt treatment initiation [30]. Data from a large prospective cohort with age-stratified clinical classification demonstrate that levels of plasma VLCFA-containing lipids, including C26:0-lysophosphatidylcholine (LPC(26:0)), correlate with disease severity across clinical phenotypes, including cerebral ALD, adrenal insufficiency, and spinal cord disease, offering potential as biomarkers for risk stratification and disease monitoring [31]. Lipidomic profiling of cerebrospinal fluid (CSF) from pediatric cerebral ALD patients identified VLCFA-containing sulfatides and phosphatidylcholines as candidate disease progression biomarkers, reflecting the compositional similarity between CSF and central nervous system tissue [32]. Consistent with this, data from newborn screening cohorts show that higher LPC(26:0) levels at birth correlate with the likelihood of developing disease manifestations in early childhood [33, 34], prompting proposals for risk-stratified surveillance protocols in which LPC(26:0) levels could be used to stratify patients as lower-risk or at-risk for developing adrenal insufficiency and cerebral ALD [35]. In contrast, earlier studies using conventional plasma VLCFA analysis failed to identify correlations with disease severity [10, 36, 37], likely because their cross-sectional designs lacked age-stratified patient classification. These designs also failed to account for the progressive nature of ALD. Patients included at the time of blood sampling may have developed additional disease manifestations in subsequent years.

Although ALD is an X-linked disorder and women were historically considered to be asymptomatic carriers, it is now well established that women can also be affected [38, 39]. Over 80% of women with ALD will develop a slowly progressive spinal cord disease, typically manifesting in the fifth decade and progressing more slowly than in men [38, 39]. Cerebral ALD and adrenal insufficiency are exceedingly rare in women, with only a few isolated cases of cerebral involvement reported [31, 40–45]. The available data suggest that cerebral involvement in women is invariably associated with extreme skewing of X-inactivation, which results in predominant expression of the pathogenic *ABCD1* allele [31, 44]. This is further supported by the strong correlation between the LPC(26:0) levels and X-inactivation patterns in female ALD patients. Greater skewing is associated with higher VLCFA-lipid levels and more severe disease manifestations [31]. Similarly, adrenal insufficiency appears to be uncommon, with overt dysfunction documented in only a few heterozygous women [46, 47].

Understanding this broad and variable clinical spectrum requires insight into the underlying biochemistry of VLCFA metabolism and how its dysregulation affects different tissues. The following sections address VLCFA homeostasis, its regulation, and its downstream pathological consequences in the brain, adrenal gland and spinal cord.

## VLCFA and VLCFA-containing lipids

Evidence for a metabolic basis of ALD first emerged from pathological and biochemical studies conducted in the early 1970s. Electron microscopy revealed characteristic lamellar lipid inclusions in adrenocortical cells. Similar inclusions were subsequently identified in testicular cells, Schwann cells, and brain macrophages [48, 49]. The unusual solubility properties of this striated material - resistant to acetone and ethanol, yet soluble in nonpolar solvents - suggested the accumulation of an exceptionally nonpolar substance [50]. Biochemical analysis showed that these inclusion bodies contained cholesterol esters and gangliosides esterified with saturated VLCFAs [51]. This established VLCFA accumulation as the biochemical hallmark of ALD and led to the development of a plasma VLCFA diagnostic test in 1981 [52].

The accumulation of VLCFAs in ALD primarily results from endogenous synthesis [53, 54] and is only minimally influenced by dietary intake [55]. The initial synthesis of long-chain fatty acids (C16 and C18) is catalyzed by fatty acid synthase in the cytosol. Subsequent elongation to VLCFAs on the cytoplasmic face of the endoplasmic reticulum proceeds via a four-step cycle of condensation, reduction, dehydration, and further reduction. The initial condensation, catalyzed by elongation of very long-chain fatty acids (ELOVL) proteins, is the rate-limiting step [56]. Seven ELOVL enzymes have been identified [57], each with specific substrate preferences [58, 59].

Once synthesized, VLCFAs must undergo thioesterification to form their coenzyme A derivatives before they can be metabolically processed. Fatty acid transport protein 4 (FATP4, encoded by *SLC27A4*) predominantly mediates this activation [60]. The resulting VLCFA-CoA esters are then translocated across the peroxisomal membrane by the ABCD1 protein, which functions as a homodimer [6]. Within the peroxisome, VLCFAs undergo β-oxidation through the sequential action of acyl-CoA oxidase 1 (ACOX1), the D-bifunctional protein (DBP, encoded by *HSD17B4*), and either the thiolase 1 (ACAA1) or the sterol carrier protein 2 (SCP2) [61]. ABCD1 protein deficiency in ALD has two major consequences: impaired peroxisomal VLCFA β-oxidation and elevated cytosolic VLCFA-CoA levels [8, 59]. These elevated cytosolic VLCFA-CoA esters serve as substrates for further elongation by ELOVL1, resulting in the accumulation of even longer-chain species [58, 59].

The exact role of VLCFA accumulation in ALD pathology remains unclear. VLCFAs are extremely hydrophobic and have physiological properties distinct from those of long-chain fatty acids, significantly affecting their behavior within cellular membranes and metabolic processes [62]. Atomistic molecular dynamics simulations have provided molecular-level insight into these membrane perturbations, showing that LPC(24:0) and LPC(26:0) disrupt sphingomyelin bilayer organization and reduce membrane fluidity through mechanisms distinct from those of shorter-chain lipids [63]. VLCFA accumulation has been linked to several adverse effects, including oxidative stress, mitochondrial dysfunction, disruption of intracellular calcium homeostasis, and an increased endoplasmic reticulum (ER) stress response [64–68]. However, it is unclear whether these effects are directly caused by VLCFA accumulation or represent secondary consequences of cellular stress responses to as-yet-unknown primary insults. The initial cellular insult in VLCFA-mediated pathology, and the basis of cell-specific vulnerability, remain to be defined.

Lipidomics studies have substantially expanded our understanding of the extent to which VLCFAs are incorporated into complex lipids in ALD. Four-dimensional (4D) lipidomics profiling identified over 1,100 lipid species in ALD fibroblasts, with significant elevations across multiple lipid classes, including lysophosphatidylcholine (LPC), phosphatidylcholine (PC), triacylglycerol (TG), sphingomyelin (SM), and cholesterol esters (CE) [69]. VLCFAs were detected in species containing up to 32 carbon atoms in their acyl chains. Targeted glycosphingolipids analysis of ALD fibroblasts revealed elevated levels of VLCFA-containing hexosylceramide and SM species [70]. Similar results were obtained in plasma from a large prospective cohort of 92 male and 65 female ALD patients, in which VLCFA incorporation was observed across LPC, PC, TG, CE, and other lipid classes, with higher levels strongly associated with disease severity across all major clinical phenotypes [31]. Lipidomic profiling of ALD-derived induced pluripotent stem cell (hiPSC)-astrocytes revealed a comparably altered lipid profile, with the largest increases in VLCFA-containing TG and CE, followed by PC and LPC. Meanwhile, the metabolome showed no major differences, suggesting that lipid dysfunction is a primary, cell-autonomous feature of ALD astrocytes [71]. Ceramide profiling of ALD plasma revealed that C26:0 ceramide is elevated across all clinical phenotypes, while C26:1 ceramide is elevated specifically in patients with myelopathy, not cerebral ALD, suggesting that ceramide species may serve as biomarkers that distinguish phenotypes [72]. Together, these data demonstrate that VLCFA accumulation in ALD profoundly alters the lipidome across multiple lipid classes and cell types, with consequences extending well beyond the originally identified cholesterol ester and ganglioside fractions (Table 1).

**Fig. 1:**
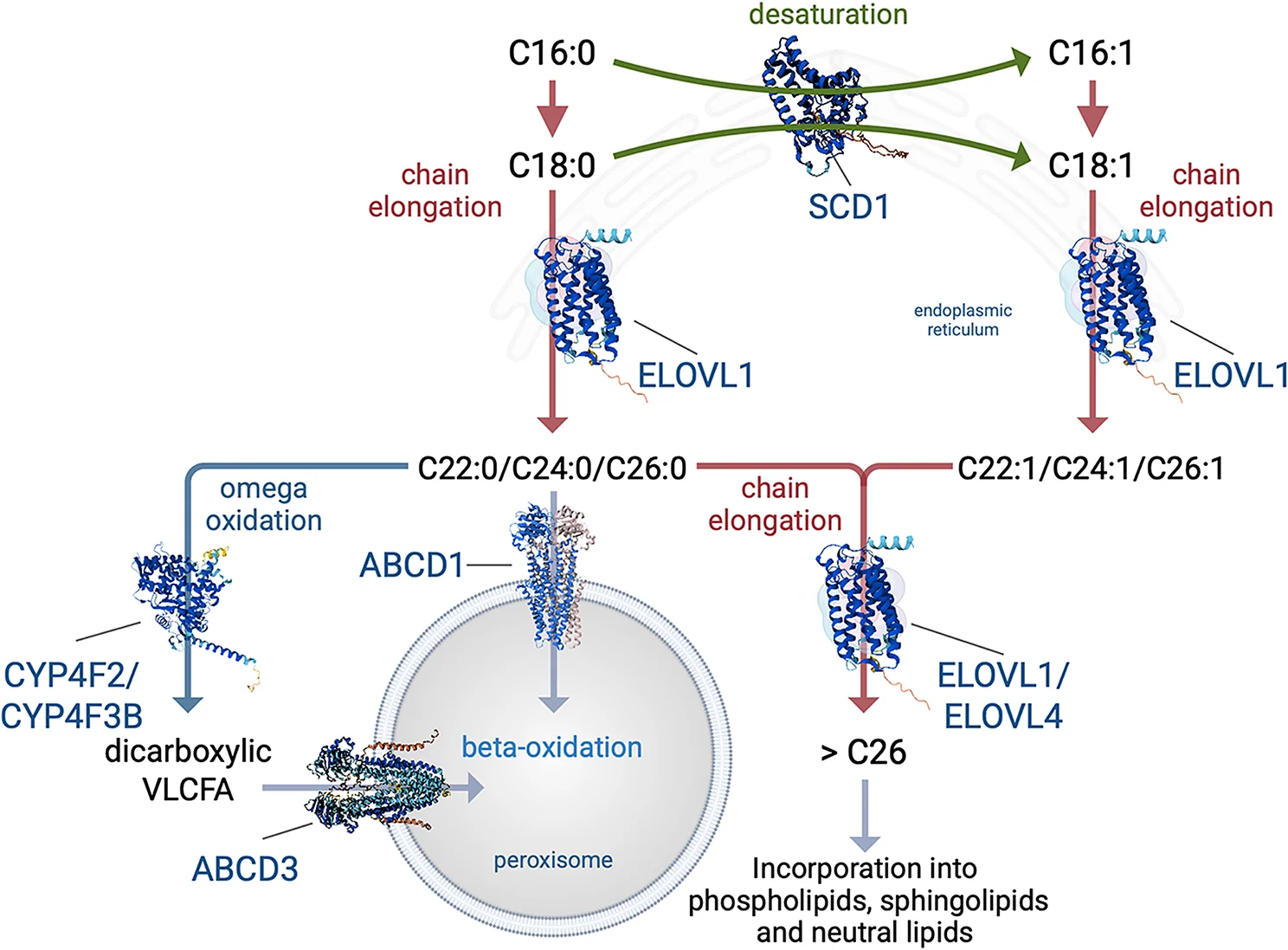
Key enzymes in VLCFA homeostasis. Very long-chain fatty acids (VLCFAs) are synthesized through the elongation of long-chain fatty acids by ELOVL1, the human VLCFA-specific elongase. Stearoyl-CoA desaturase-1 (SCD1) catalyzes the conversion of saturated long-chain fatty acids into monounsaturated fatty acids, thereby reducing the availability of saturated fatty acid precursors for VLCFA synthesis. ABCD1 deficiency impairs the import of VLCFAs into peroxisomes and their subsequent β-oxidation. This leads to an increase in cytosolic VLCFA levels, which results in further fatty acid chain elongation by ELOVL1 and ELOVL4 and enhanced incorporation of VLCFAs into complex lipids. ω-oxidation by CYP4F2/CYP4F3B provides a possible escape route, as dicarboxylic VLCFAs are transported into peroxisomes via ABCD3. Predicted protein structures are from the AlphaFold EMBL-EBI protein structure database [78, 79] Created in BioRender. Kemp, S. (2026) https://BioRender.com/dzq1ysb

VLCFAs and VLCFA-containing lipid levels in ALD are influenced by several metabolic processes (Fig. 1). These include the availability of fatty acids for VLCFA synthesis, the rate of VLCFA synthesis, residual ABCD1-mediated peroxisomal membrane translocation capacity, peroxisomal β-oxidation, and VLCFA elongation to longer species by ELOVL1 [58, 59]. C26 fatty acids can be further elongated to ultra-long-chain fatty acids (≥ C28) by ELOVL4 [73]. In addition, dicarboxylic-VLCFA formation via ω-oxidation by CYP4F2 and CYP4F3B [74, 75], followed by transport into peroxisomes by ABCD3 [76], and VLCFA incorporation into complex lipids also influence VLCFA levels in ALD [58, 77].

**Table 1:** Major VLCFA-containing lipid classes in ALD: tissue distribution, pathological roles, and biomarker potential

Lipid class	Change in ALD	Tissue/biofluid	Pathological mechanism	Reference
LPC(26:0)	↑	Plasma	Microglial cytotoxicity; correlates with disease severity across all phenotypes; basis for risk stratification	[31, 35, 80]
PC(VLCFA)	↑	Brain white matter, CSF	Membrane disruption; detectable prior to overt demyelination	[81–83]
CE(VLCFA)	↑	Brain, adrenal gland, fibroblasts, astrocytes	Neuroinflammatory cascades; cholesterol sequestration impairing steroidogenesis	[31, 51, 71], 83– [85]
TG(VLCFA)	↑	Plasma, astrocytes	Lipid droplet accumulation; possible protective sequestration or toxic lipid particle secretion	[31, 71]
SM(VLCFA)	↑	Plasma, fibroblasts	Altered membrane lipid composition	[69, 70, 83]
Ceramide C26:0	↑	Plasma	Elevated across all clinical phenotypes	[72]
Ceramide C26:1	↑	Plasma	Elevated specifically in myelopathy; candidate phenotype-distinguishing biomarker	[72]
PE plasmalogen	↓	Brain, spinal cord	Oxidative stress marker; enhanced phospholipase A2-mediated degradation	[83, 85, 86]
Sulfatides, GM1	↓	Brain	Impaired myelin maintenance; depletion despite stable oligodendrocyte population suggests lipid-driven dysfunction	[85]

## Regulation and therapeutic targets of VLCFA homeostasis

Changes in the enzymatic activity of key enzymes involved in these metabolic pathways directly impact VLCFA homeostasis and levels, and several represent potential therapeutic targets. Pharmacological activation of stearoyl-CoA desaturase-1 (SCD1), the enzyme that catalyzes the conversion of saturated long-chain fatty acids (C16:0 and C18:0) to their monounsaturated counterparts [87], reduces *de novo* synthesis of saturated VLCFAs by redirecting elongation substrates toward monounsaturated species. This decreases the availability of saturated precursors and lowers C26:0 levels in vitro and in Abcd1-deficient mice [64]. Conversely, SCD1 inhibition increased C26:0 levels [64]. However, SCD1 is a central lipogenic regulator with well-established roles in fatty acid metabolism, obesity, and tumorigenesis [88, 89]. Therefore, chronic pharmacological activation of SCD1 as a therapeutic strategy will require careful evaluation of systemic metabolic risks.

VLCFA synthesis primarily depends on long-chain fatty acid elongation, which is mainly regulated by ELOVL1 [58, 59]. Pharmacological inhibition of ELOVL1 reduces C26:0 synthesis and levels [90–93]. Bezafibrate, a fibrate drug used for dyslipidemia, was the first drug demonstrated to lower VLCFA levels in ALD fibroblasts by directly inhibiting ELOVL1 elongation activity, independently of its known peroxisome proliferator-activated receptor (PPAR) activation effects [90]. However, a subsequent proof-of-principle clinical trial in ten ALD patients (BEZA trial, NCT01165060) showed no reduction in plasma or lymphocyte VLCFA levels at doses up to 800 mg per day. Plasma bezafibrate concentrations did not exceed 25 µmol/L, which is far below the ~ 400 µmol/L required for maximal ELOVL1 inhibition in vitro. This indicates that inadequate intracellular drug concentrations precluded therapeutic efficacy in vivo [94]. These findings validated ELOVL1 inhibition as a concept, but also identified the need for more potent, CNS-penetrant compounds. This motivated the subsequent development of selective ELOVL1 inhibitors with superior pharmacological properties [92, 93]. In vivo validation was achieved when an ELOVL1 inhibitor significantly reduced C26:0 VLCFA levels in the brains and spinal cords of Abcd1 knockout mice, demonstrating CNS penetrance and supporting ELOVL1 inhibition as a viable substrate-reduction strategy for ALD [95]. Blood LPC(26:0) levels closely tracked this response and normalized rapidly upon ELOVL1 inhibition. This finding supports the use of LPC(26:0) as a pharmacodynamic biomarker for monitoring systemic biochemical correction in future clinical trials [96]. However, achieving meaningful VLCFA reduction in the CNS requires prolonged and potent inhibition because sphingolipids, a major subclass of CNS lipids, turn over slowly and are substantially less responsive to ELOVL1 inhibition than phosphatidylcholines in treated mice [95]. Preclinical safety studies across multiple ELOVL1 inhibitor series identified dose-limiting toxicities in the skin, gastrointestinal tract, and ocular tissues. Single-nucleus RNA sequencing revealed the unexpected activation of protein unfolding stress and ERAD signaling in spinal cord neurons upon ELOVL1 inhibition. These findings may reflect on-target effects and highlight the need for careful optimization of the therapeutic index before clinical translation [92, 95]. The importance of maintaining adequate VLCFA levels is underscored by the observation that complete loss of ELOVL1 function is pathogenic. Homozygous Elovl1 knockout mice die as neonates due to a defective skin barrier [77]. Patients with dominant or homozygous ELOVL1 pathogenic mutations exhibit ichthyosis, hypomyelination, and spastic paraplegia, associated with subnormal VLCFA levels [97, 98]. These observations suggest that the therapeutic window for ELOVL1 inhibition lies between the pathologically elevated VLCFA levels associated with ALD and the subnormal levels associated with ELOVL1 deficiency. Defining the optimal degree of ELOVL1 inhibition, sufficient to meaningfully reduce VLCFA levels without depleting them below physiological thresholds, will require careful dose-ranging studies in preclinical models and ultimately, clinical studies.

Similarly, genetic variants in key enzymes involved in VLCFA homeostasis can influence VLCFA levels. For example, the p.V433M variant of CYP4F2 decreases enzyme levels and impairs the conversion of VLCFAs to VLCFA dicarboxylic acids via ω-oxidation [99]. The ω-oxidation pathway involves the conversion of VLCFAs to ω-hydroxy-VLCFAs and then to dicarboxylic VLCFAs via ALDH3A2-dependent and alternative CYP4F-mediated routes. This pathway is independent of the ABCD1 protein [75, 100]. Dicarboxylic acids are transported into the peroxisome by the ABCD3 protein [76]. CYP4F2 expression can be induced by statins through activation of the sterol regulatory element-binding protein 2 (SREBP-2) pathway, and CYP4F3 is inducible by all-trans-retinoic acid, providing pharmacological approaches to upregulate ω-oxidation capacity as a therapeutic strategy [101, 102].

Dietary supplementation with monounsaturated fatty acids has also been explored as a way to redirect elongation flux away from the synthesis of saturated VLCFAs. Lorenzo’s oil, a 4:1 mixture of oleic acid (C18:1) and erucic acid (C22:1), normalizes plasma C26:0 levels within weeks of initiation [103]. These monounsaturated fatty acids act as competing substrates for elongation by ELOVL1, thereby reducing saturated VLCFA synthesis [104]. However, normalization of VLCFAs is restricted to plasma. It remains unclear whether Lorenzo’s oil meaningfully reduces VLCFA levels in tissues, including the central nervous system, as direct measurements are absent from the literature and Lorenzo’s oil does not cross the blood-brain barrier [105]. Consistent with this limitation, randomized and open-label trials have not demonstrated neurological or endocrine benefits or arrest of disease progression [106–108]. A large prospective cohort study of presymptomatic boys with normal MRIs suggested that Lorenzo’s oil might reduce the risk of developing cerebral ALD during childhood [109]. However, this study lacked a concurrent placebo-controlled group and relied on historical estimates of disease incidence. Therefore, the preventive effect of Lorenzo’s oil remains uncertain. Furthermore, Lorenzo’s oil therapy is associated with adverse effects, including elevated liver enzymes and thrombocytopenia [108], and it is no longer routinely reimbursed or recommended as the standard of care. These limitations have prompted investigation of alternative dietary strategies that may achieve more substantial VLCFA reduction, including within the central nervous system. Nervonic acid (C24:1), a monounsaturated VLCFA that is naturally enriched in myelin, has been investigated for this purpose. In Abcd1 knockout mice, nervonic acid supplementation reduced plasma LPC(26:0) by approximately 60% within one week and brain LPC(26:0) by approximately 56% after four weeks [110]. These results suggest that nervonic acid may penetrate the central nervous system more effectively than Lorenzo’s oil. However, direct evidence of brain penetration and efficacy in humans is still lacking. More broadly, dietary fatty acid interventions, such as Lorenzo’s oil and nervonic acid, alter systemic lipid composition in a relatively broad and nonspecific manner. This has wider effects on fatty acid elongation, membrane composition, and other lipid-dependent pathways that remain incompletely understood. Selective small-molecule ELOVL1 inhibitors may therefore offer a more targeted and pharmacologically controllable approach to reducing saturated VLCFA synthesis.

Together, these enzymatic and dietary VLCFA-lowering approaches are complementary to therapies that restore ABCD1 function, such as hematopoietic stem cell transplantation and gene therapy. Although these therapies only partially normalize VLCFA levels [64, 95], complete biochemical correction may not be necessary for clinical benefit, as suggested by the nearly exclusive occurrence of cerebral ALD and adrenal insufficiency in women with extreme X-inactivation skewing toward the mutant allele [31], implying that partial preservation of ABCD1 function, and by extension partial VLCFA reduction, may prevent severe disease manifestations.

## Tissue-specific consequences of VLCFA accumulation

### Cerebral ALD

In the brain, the accumulation of VLCFA in specific lipid classes appears to play a central role in the pathogenesis of cerebral ALD. The following section addresses this from three interconnected angles: VLCFA incorporation into membrane phospholipids as a structural insult, the resulting inflammatory cascades, and downstream oxidative stress and plasmalogen depletion.

## VLCFA in membrane phospholipids: structural disruption

Several studies have shown that VLCFAs in the brain are predominantly located in phospholipids, which may be an important factor in the development of cerebral ALD [81, 111]. This is supported by the demonstration that incorporation of VLCFAs into artificial phospholipid vesicles resulted in disruption of membrane structure and function [62]. Analysis of the lipid profile in postmortem brains from cerebral ALD cases revealed a significant increase in VLCFA-containing cholesterol esters in active demyelinating areas. Notably, in both the active demyelinating area and the intact white matter, VLCFAs were prominently enriched in the PC fraction [81], indicating that VLCFA enrichment in PC is already present in white matter prior to overt demyelination. A comprehensive analysis of the lipid classes in ALD brain tissue showed that C26:0 levels are elevated across all major lipid classes in white matter, including SM, cerebrosides, sulfatides, and PC, whereas in active plaque tissue the majority of C26:0 is concentrated in CE, which is consistent with the progressive sequestration of VLCFAs into CE during demyelination [83]. Elevated levels of VLCFA-containing PC have also been reported in the CSF of two ALD patients [82]. In addition, VLCFA-enriched LPCs are cytotoxic, as intracortical injection of LPC(24:0) induced widespread microglial activation and apoptosis in wild-type mice [80]. Importantly, this effect was absent with LPC(16:0) injection, underscoring that saturated VLCFA incorporation into lipids results in enrichment of cytotoxic lipid variants. Decreased serum levels of LPC(20:3) and LPC(20:4) have been reported in cerebral ALD patients compared to asymptomatic ALD patients [112].

In patients with cerebral ALD, VLCFAs and VLCFA-containing lipids are more prevalent in regions of inflammation and plaques than in normal-appearing white matter [113–115]. Moreover, the presence of VLCFAs in normal-appearing white matter is more prevalent in patients with cerebral ALD than in those without cerebral involvement [116]. Additionally, lower levels of the monounsaturated fatty acids C22:1 and C24:1 were found in patients with cerebral ALD [116]. High levels of saturated VLCFAs induce oxidative stress, mitochondrial dysfunction, and an increased endoplasmic reticulum (ER) stress response [64–68]. In contrast, monounsaturated VLCFAs do not cause these effects [64, 65] and may even attenuate ER stress induced by high levels of saturated VLCFAs [117]. These differential effects of saturated versus monounsaturated VLCFAs are consistent with findings in ALD-derived iPSC-astrocytes, where exposure to C26:0 reduced cell viability, whereas exposure to C26:1 did not [71]. They are also consistent with lipidomics data showing that fold changes of elevated lipid species in ALD increase with chain length and degree of saturation [31]. Further evidence of the neurotoxic potential of VLCFA-containing lipids comes from studies in non-ALD neuroinflammatory contexts. Reactive astrocytes upregulate VLCFA-containing phosphatidylcholines and long-chain saturated free fatty acids. These are secreted via APOE- and APOJ-containing lipoparticles, which mediate lipoapoptotic death of neurons and oligodendrocytes through the PERK/PUMA pathway [118]. Astrocyte-specific knockout of ELOVL1 significantly reduced this lipid-mediated neurotoxicity, directly implicating ELOVL1-synthesized saturated lipids as mediators of glial neurotoxicity [118]. These findings are directly relevant to ALD, in which VLCFA accumulation drives astrocyte reactivity and VLCFA-containing phosphatidylcholines accumulate in white matter.

## Cholesterol ester accumulation

Approximately 1% of brain cholesterol is stored as CE in lipid droplets synthesized by sterol O-acyltransferase (SOAT1), also referred to as acyl-CoA cholesterol acyltransferase 1 (ACAT1) [119]. Increased SOAT1 activity has been associated with inflammatory conditions and is considered a potential therapeutic target in the treatment of atherosclerosis and Alzheimer’s disease [119–121].

ABCD1 deficiency leads to sequestration of cholesterol in VLCFA-containing CE, reducing the availability of free cholesterol [84]. Consistent with this, studies of the lipidome in ALD fibroblasts, astrocytes, and cortical organoids have identified significant increases in VLCFA-containing CE. This suggests that CE accumulation is a consistent, cross-tissue feature of the ALD lipidome [31, 71, 85]. The link between VLCFA-driven CE accumulation and neuroinflammation in ALD is further illustrated by the finding that ABCD1 deficiency promotes the abnormal expression of cholesterol 25-hydroxylase (CH25H) and subsequent accumulation of 25-hydroxycholesterol (25-HC) [122]. In ALD iPSC-derived cells, 25-HC, rather than VLCFAs, robustly activates the NLRP3 inflammasome through K⁺ efflux, mitochondrial ROS, and LXR-mediated pathways. This leads to microglial recruitment, IL-1β production, and oligodendrocyte death in the corpus callosum [122]. NLRP3 inflammasome activation has since been confirmed in postmortem human cerebral ALD tissue [123].

### Inflammatory cascades: arachidonic acid and leukotrienes

Arachidonic acid (AA) is an omega-6 polyunsaturated fatty acid that is essential for brain function. AA is highly abundant in the CNS, particularly in neurons, and plays a critical role in signaling pathways that regulate inflammation, neuroplasticity, and synaptic function. AA metabolism has been implicated in the pathogenesis of cerebral ALD [113]. AA can be metabolized by 5-lipoxygenase (5-LOX) to leukotrienes, which act as chemoattractants and contribute to inflammatory cell death. The levels of AA, leukotriene B4 (LTB4), and cysteinyl leukotrienes are elevated in all areas of the cerebral ALD brain compared to control brains, with the highest levels observed in plaques and plaque shadows [113]. LTB4 levels are also elevated in the CSF of cerebral ALD patients compared to ALD patients without cerebral involvement [124]. Furthermore, 5-LOX and cPLA2 are elevated in cerebral ALD brain, with the highest levels observed in the plaque area and plaque shadow [113]. Consistent with this, in cultured cells, elevated VLCFA increased the expression of 5-LOX, suggesting that VLCFA accumulation may enhance 5-LOX activity [113]. This aligns with earlier work showing that VLCFA accumulation correlates with increased expression of inflammatory mediators and disease progression in cerebral ALD [114]. Several of these inflammatory mediators, such as IL-1β, TNF-α, and IL-6, can be upregulated by AA-derived eicosanoids, including prostaglandins and leukotrienes. In addition, F2-isoprostanes, oxygenated metabolites of AA, are elevated in the plasma of ALD patients and are associated with cerebral ALD [125]. AA is also a precursor for inflammatory mediators such as malondialdehyde, 4-hydroxynonenal, and acrolein, which have been documented in cerebral ALD brain [86, 113, 126].

In line with the idea that saturated VLCFAs play a role in driving innate immune activation, transcriptome-wide profiling of macrophages loaded with C24:0 and C26:0 at ALD-relevant concentrations revealed proinflammatory skewing and upregulation of chemotaxis and invasion factors [127]. Externally added VLCFAs were found to enhance monocyte chemotaxis and invasive capacity in vitro [127]. These findings suggest that VLCFA-driven macrophage activation contributes to the neuroinflammatory cascade in cerebral ALD. Additionally, VLCFA accumulation in glia may drive neuroinflammation by producing sphingosine-1-phosphate (S1P). Glial cells convert VLCFAs into S1P via VLCFA-containing ceramide intermediates. Excess S1P activates NF-κB signaling and promotes the infiltration of macrophages into the CNS, a pathway that can be attenuated by S1P receptor antagonism [128].

Pathological studies of cerebral ALD lesions reveal a striking spatial pattern of microglial behavior directly linked to VLCFA toxicity. Microglia increase in number in normal-appearing white matter, undergo programmed cell death in the prelesional zone (preceding major oligodendrocyte and myelin degeneration), and are virtually absent in the actively demyelinating zone [129]. They then return to a homeostatic phenotype in the gliotic core. This sequence suggests that VLCFA-rich myelin debris shed by oligodendrocytes is initially sequestered by microglia, which subsequently fail to degrade it and undergo apoptosis, releasing their contents into surrounding tissue and thereby propagating the inflammatory cascade [129]. The cytotoxicity of VLCFA-containing LPC to microglia has been directly demonstrated by intracortical injection of LPC(24:0) in mice, which reproduced the apoptosis and spatial distribution pattern of microglia characteristic of ALD lesions [80]. Importantly, a detailed comparison of axonal and myelin pathology in cerebral ALD and multiple sclerosis (MS) autopsy tissue demonstrated that, in contrast to MS, acute axonal damage in cerebral ALD occurs in prelesional areas defined by microglial loss and is not dependent on inflammatory cell infiltration. Unlike MS, which exhibits selective myelin phagocytosis, cerebral ALD lesions at all stages demonstrate concurrent and nonselective destruction of the entire axon-myelin unit. Although regenerating oligodendrocytes indicate that remyelination pathways are activated, these repair attempts appear to be hindered by the ongoing disease process [130]. This pattern implicates the loss of myeloid cell function as an early checkpoint in lesion formation that increases the metabolic burden on surrounding tissue and contributes to axonal demise independently of damage caused by inflammatory bystander effects.

## Oxidative stress and plasmalogen depletion

Reduced levels of ether-linked PE have been observed in brain tissue from ALD patients and Abcd1 knockout mice [83, 86]. This reduction has been linked to increased oxidative stress, as demonstrated by elevated levels of reactive aldehydes and oxidized proteins [86]. Similar plasmalogen reductions have been reported in brain tissue from Alzheimer’s disease patients, where reduced PE plasmalogen levels are associated with neurofibrillary tangle pathology [131, 132]. Evidence from human iPSC-derived models further supports these findings: lipidomic profiling of ALD spinal cord organoids revealed decreased levels of PE plasmalogens alongside increased lysophospholipid derivatives (LPE, LPE[O], and LPE[P]) [85]. In ALD cortical organoids, reductions in gangliosides (GM1) and sulfatides were observed, consistent with earlier reports from postmortem ALD white matter [85]. Since sulfatides and gangliosides are critical for myelin integrity and stabilizing axoglial junction proteins, such as myelin-associated glycoprotein (MAG) and neurofascin 155 (NF155), their depletion, suggests lipid-driven impairment of myelin maintenance. Ferroptosis, an iron-dependent form of cell death driven by lipid peroxidation, has been associated with the pathogenesis of cerebral ALD [133]. Quantitative susceptibility mapping has revealed abnormal iron accumulation in cerebral ALD lesions and perilesional white matter, with co-localization of lipid peroxidation markers and microglial loss [133]. These findings suggest that VLCFA-driven oxidative stress may converge on ferroptotic cell death pathways. Furthermore, fibroblasts from cerebral and non-cerebral ALD patients exhibit distinct oxidative stress responses. Compared with controls and cerebral ALD fibroblasts, non-cerebral ALD fibroblasts show significantly lower oxygen consumption rates and reduced basal expression of NRF2-dependent antioxidant genes, including NQO1, AKR1B10, and AKR1C1. Moreover, when exposed to additional oxidative stress, non-cerebral ALD fibroblasts demonstrate greater compensatory capacity. This suggests that variations in oxidative stress responses may contribute to phenotypic differences in ALD [134].

## Adrenal insufficiency

The exact mechanism by which VLCFA accumulation affects adrenal gland function remains unclear. VLCFAs accumulate preferentially in the zona fasciculata and zona reticularis of the adrenal cortex, while the zona glomerulosa is relatively spared [48, 49]. Since the zona fasciculata and reticularis primarily produce cortisol and androgens, respectively, deficiencies in glucocorticoids and androgens are more common manifestations of adrenal disease in ALD than mineralocorticoid deficiency [135]. For reasons that remain unclear, the zona reticularis appears to be more resistant to VLCFA-mediated toxicity than the zona fasciculata. This may reflect zone-specific differences in lipid metabolism, cholesterol trafficking, or mitochondrial function. It has been suggested that VLCFA accumulation in CE leads to apoptosis and atrophy of the adrenal cortex, thereby disrupting cortisol production [136]. When VLCFAs were added to human adrenocortical cell cultures at concentrations similar to those found in ALD plasma, membrane microviscosity increased and ACTH-stimulated cortisol secretion decreased, suggesting that excess VLCFAs alter membrane structure and interfere with ACTH receptor availability [137]. An additional mechanism involves the sequestration of cholesterol into VLCFA-containing CE within lipid droplets, reducing free cholesterol availability as a substrate for *de novo* steroidogenesis. Consistent with this, progesterone-induced lipolysis of CE-rich lipid droplets significantly increased cortisol secretion from ALD fibroblasts compared to controls, suggesting that cholesterol trapped in VLCFA-CE restricts steroidogenic capacity [84]. Similar lamellar inclusions are found in testicular Leydig cells, which undergo the same VLCFA-mediated toxic atrophy as adrenocortical cells [48, 138]. This may result in impaired testosterone production, and biochemical hypogonadism occurs in approximately 11% of male patients [139]. Plasma levels of VLCFA-containing lipids, including LPC(26:0), are significantly higher in ALD patients with adrenal insufficiency than in those without, supporting a direct relationship between systemic VLCFA lipid burden and adrenocortical dysfunction [31]. Together, these data suggest that VLCFA-mediated disruption of adrenocortical structure and function underlies the high prevalence of adrenal insufficiency in ALD. This process begins in utero, as demonstrated by pathological lesions in fetal adrenal tissue [140], and adrenal insufficiency ultimately develops in 80–86% of males, peaking between ages three and ten [14, 141]. Newborn screening has shown that biochemical evidence of adrenal insufficiency can appear as early as five weeks of age [142]. Despite its high prevalence, adrenal insufficiency is often overlooked or diagnosed late, with an average delay of 3.5 years reported between symptom onset and diagnosis [14]. HCT does not prevent or reverse adrenal insufficiency, as bone marrow-derived cells do not migrate to the adrenal cortex. Unlike in cerebral ALD, where donor-derived microglia can arrest neuroinflammation, adrenocortical cells continue to accumulate VLCFA regardless of transplantation status [17, 143]. Unlike patients with autoimmune adrenal failure (Addison’s disease), most patients with ALD are not mineralocorticoid deficient. This is consistent with the relative sparing of the zona glomerulosa [48, 49].

## Spinal cord disease

Spinal cord disease is the most prevalent clinical manifestation of ALD and the primary cause of long-term disability in affected males and females [18, 38, 39]. Nearly all male patients develop progressive myelopathy, with symptoms typically beginning in the third decade of life and progressing to the point where they need a walking aid or a wheelchair in the sixth decade [18, 144]. In women, myelopathy follows a similar, albeit slower, course, with symptoms typically appearing in the fifth decade of life [38, 39]. Currently, there is no disease-modifying treatment available.

The neuropathological hallmark of ALD-related spinal cord disease is a dying-back axonopathy that predominantly affects the long ascending and descending tracts, particularly the fasciculus gracilis and lateral corticospinal tracts, in a bilateral and largely symmetrical pattern [138]. Within affected tracts axonal and myelin loss occur in parallel. Microglia predominate while lymphocytes are sparse to absent. This underscores the non-inflammatory nature of this myelopathy, in contrast to the inflammatory changes seen in cerebral ALD [12, 138]. An abnormal concentration of VLCFAs in myelin lipids of the spinal cord is associated with oxidative stress and impaired mitochondrial function [66]. These factors likely contribute to axonal dysfunction by disrupting ATP-dependent axonal transport. It has been proposed that VLCFAs incorporated into gangliosides disrupt the axonal membrane microenvironment, leading to dysfunction and atrophy [138]. Lumbar dorsal root ganglia from ALD patients exhibit neuronal atrophy, and mitochondria-containing lipid inclusions within neurons suggest impaired mitochondrial function [145]. Elevated phagocytosis markers, including MFGE8 and TREM2, precede synapse loss in the spinal cords of ALD patients. Adding LPC(26:0) to ABCD1-deficient microglia increased MFGE8 expression, exacerbating phagocytosis and neuronal injury [146]. These findings implicate VLCFA-containing lipids in spinal cord pathology, consistent with lipidomic data showing that plasma LPC(26:0) levels correlate with myelopathy severity in male ALD patients, suggesting a dose-dependent relationship between systemic VLCFA lipid burden and spinal cord neurodegeneration [31].

## Conclusions

Despite their distinct clinical manifestations, cerebral ALD, spinal cord disease, and adrenal insufficiency share a common biochemical origin: impaired ABCD1-dependent peroxisomal handling of VLCFAs and subsequent accumulation of VLCFA-containing complex lipids. In affected tissues, these lipids are incorporated into phospholipids, sphingolipids, cholesterol esters, and other lipid classes. There, they disrupt membrane organization, lipid trafficking, and organelle function while activating cellular stress responses. In the brain, this results in a self-amplifying inflammatory cascade that leads to leukodystrophy. In the spinal cord, it drives a non-inflammatory dying-back axonopathy. In the adrenal gland, VLCFA accumulation progressively disrupts steroidogenic capacity through structural atrophy and cholesterol sequestration.

The tissue-specific nature of these outcomes remains incompletely understood. Patients with identical pathogenic *ABCD1* variants can exhibit markedly different phenotypes. Differences in lipid class incorporation, acyl-chain length and saturation, lipid turnover, cellular stress responses, and tissue-specific repair capacity likely determine vulnerability. Lipidomics studies support this view, showing that VLCFA-containing lipid species differ across tissues and phenotypes. Generally, saturated and longer-chain species show the strongest associations with toxicity and disease severity. Secondary lipid mediators, including VLCFA-containing LPC, cholesterol esters, 25-hydroxycholesterol, and oxidized lipid products, may further amplify primary VLCFA toxicity through cell-type-specific inflammatory, oxidative, and metabolic pathways.

The emergence of LPC(26:0) and other VLCFA-containing lipid species as biomarkers that correlate with disease severity across all three tissues suggests that the systemic lipidome reflects and may predict tissue-specific disease burden. Thus, comprehensive lipidomic profiling is positioned not only as a research tool, but also as a potential clinical instrument for risk stratification and treatment monitoring.

Understanding these shared and tissue-specific mechanisms is essential for developing substrate-reduction strategies that target VLCFA homeostasis across the full clinical spectrum of ALD. The nearly exclusive occurrence of cerebral ALD and adrenal insufficiency in women with high VLCFA-lipid levels due to extreme X-inactivation skewing suggests that partially reducing the VLCFA lipid burden, rather than completely normalizing it, may be sufficient to prevent severe disease manifestations. This differs mechanistically from the mode of action of HCT, which primarily arrests cerebral disease by replacing brain-resident microglia with donor-derived cells. Women with random X-inactivation carry a substantially higher proportion of ABCD1-expressing cells than HCT achieves; however, they still develop myelopathy. This observation underscores that cell replacement alone does not fully explain phenotypic protection. Furthermore, HCT does not prevent myelopathy development in adult males [147], which is consistent with the notion that spinal cord disease occurs via a distinct, non-inflammatory mechanism beyond the scope of replacing microglia. Taken together, these observations support the premise that reducing the systemic VLCFA lipid burden itself confers protection. Substrate reduction therapy targeting myelopathy will likely require a meaningful reduction in VLCFA lipid levels. Prospective deep phenotyping and biochemical profiling of female ALD cohorts across the full spectrum of X-inactivation patterns may help define the therapeutic threshold — the extent to which VLCFA levels must be lowered to meaningfully attenuate spinal cord disease progression. This would inform the design of future clinical trials.

## Data Availability

No datasets were generated or analysed during the current study.

## Abbreviations

25-HC: 25-Hydroxycholesterol
5-LOX: 5-Lipoxygenase
AA: Arachidonic acid
ACAA1: Thiolase 1 (acetyl-CoA acyltransferase 1)
ACOX1: Acyl-CoA oxidase 1
ACTH: Adrenocorticotropic hormone
AKR1B10: Aldo-keto reductase family 1 member B10
AKR1C1: Aldo-keto reductase family 1 member C1
ALD: X-linked adrenoleukodystrophy
AMN: Adrenomyeloneuropathy
APOE: Apolipoprotein E
APOJ: Apolipoprotein J
CE: Cholesterol ester
CH25H: Cholesterol 25-hydroxylase
cPLA2: Cytosolic phospholipase A2
CSF: Cerebrospinal fluid
CYP4F2: Cytochrome P450 family 4 subfamily F member 2
CYP4F3B: Cytochrome P450 family 4 subfamily F member 3B
DBP: D-bifunctional protein
ELOVL: Elongation of very long-chain fatty acids protein
ERAD: Endoplasmic reticulum-associated degradation
FATP4: Fatty acid transport protein 4
hiPSC: Human induced pluripotent stem cell
IL-1β: Interleukin-1 beta
LPC: Lysophosphatidylcholine
LPE: Lysophosphatidylethanolamine
LTB4: Leukotriene B4
LXR: Liver X receptor
MAG: Myelin-associated glycoprotein
MFGE8: Milk fat globule-EGF factor 8
MRI: Magnetic resonance imaging
MS: Multiple sclerosis
NAWM: Normal-appearing white matter
NF155: Neurofascin 155
NLRP3: NOD-, LRR- and pyrin domain-containing protein 3
NQO1: NAD(P)H quinone oxidoreductase 1
NRF2: Nuclear factor erythroid 2-related factor 2
PC: Phosphatidylcholine
PE: Phosphatidylethanolamine
PERK: Protein kinase R-like ER kinase
PPAR: Peroxisome proliferator-activated receptor
PUMA: p53 upregulated modulator of apoptosis
SCD1: Stearoyl-CoA desaturase-1
SCPx: Sterol carrier protein X
SM: Sphingomyelin
SOAT1: Sterol O-acyltransferase 1
SREBP-2: Sterol regulatory element-binding protein 2
TG: Triacylglycerol
TNF-α: Tumor necrosis factor alpha
TREM2: Triggering receptor expressed on myeloid cells 2
VLCFA: Very-long-chain fatty acids
